# On the Enamel and Dentine of the Teeth

**Published:** 1855-04

**Authors:** T. H. Huxley


### ARTICLE XVI.

### *On the Enamel and Dentine of the Teeth.* By T. H. HUXLEY, F. R. S.

THE first part of the sixth volume of Siebold and Kölliker's "Zeitschrift fur Wissenschaftliche Zoologie," published in July of the present year, contains a paper by M. Edouard Lent, "On the Development of the Dentine and Enamel of the Teeth." It could only be a source of gratification to me that any one should have been led fairly to investigate a subject, by the perusal of a paper of mine published in this Journal, even if his results were totally opposed to those at which I had arrived; and I do not think that, as a general rule, controversial writing is worth the paper it is printed on—assuredly, it is not worth the time it wastes. I should, therefore, have had nothing to reply to M. Lent's unfavorable expressions with regard to my labors, were it not for two circumstances. In the first place, M. Lent is a pupil of Professor Kölliker's, and appears to have worked under the eye of that distinguished investigator. Indeed, the paper is so completely sanctioned by Professor Kölliker, that I must regard him as responsible for it. And in the second place, owing, as I suppose, to M. Lent's inexperience as an author (though truly the superintendence of so practiced a writer as Professor Kölliker should have obviated this difficulty,) his paper is curiously inconsistent with itself, being in form a severe criticism and refutation—but, in fact, a confirmation—of the views I ventured to promulgate.

My paper was intended to establish two main points—1. That there is no evidence that the dentine is formed by direct conversion of pre-existing elements of the pulp. 2. That the enamel is not developed externally to the so-called basement membrane, or membrana preformativa of the pulp, but internally to it; Nasmyth's membrane, which lies over the enamel, being in fact continuous with the membrana preformativa.

1. On this head, M. Lent adds no new fact or argument of any kind. He simply repeats and confirms the statement already made by Professor Kölliker—that minute gelatinous processes may often be found passing from the surface of the pulp into the dentinal canals (a statement which no one denies,) and then takes for granted Professor Kölliker's purely hypothetical interpretation of this fact—*i. e.*, that these processes are outgrowths of the "cells" of the surface of the pulp—an hypothesis which is not supported, so far as I am aware, by a shadow of direct evidence; and it is to be remembered that the *fact* is as well accounted for by my hypothesis as by any other.

So much for the formation of the dentinal tubules. With regard to the substance of the dentine, M. Lent does not seem to have seen much more than myself; for he says that "it is more probable—indeed certain" that the "grund-substanz" is an "*excretion of the cells and their processes.*" (p. 127.)

That is to say, M. Lent (*i. e.* Professor Kölliker) admits that there is no new evidence to be brought forward as to the formation of the dentine tubules, and that the substance of the dentine is formed as I have stated it to be. Truly, then, I do not see where the "*irrige ansicht*" with which I am charged lies.

At page 128 there is a very careless misstatement, which one might be disposed to overlook in a student, but which is utterly unpardonable in a production which has the advantage of Professor Kölliker's deliberate *imprimatur.*

"As regards Huxley's erroneous view as to the formation of the dentine, I will only briefly remark, that Huxley has been so unfortunate as to have seen the dentine from above only." (p. 128.)

This is truly astounding, considering that at page 160 of my paper I give particular directions how to obtain a profile view of the dentine in undisturbed connection with the pulp; that figs. 3 and 4 are careful representations of such profile views; and that I lay particular stress upon the advantages to be derived from this mode of examination.

2. With regard to the development of the enamel, M. Lent

(*i. e.* Professor Kölliker) affords a confirmation of my views; all the more valuable, as it is evidently most unwillingly wrung from him.

At page 129, M. Lent, after stating the ordinary theory of the development of the enamel, says: "This simple theory must, however, I believe, be given up, since Huxley has made a discovery whose truth I must confirm—a discovery which greatly enhances the difficulty of accounting for the formation of the enamel."

Again, at page 133: "From what has been said, it is pretty clear that the membrana preformativa, which may be detached from the enamel in the fœtal tooth, subsequently becomes the so-called cuticle of the enamel, as, indeed, Huxley states."

Again, at page 130: "When, however, I tested Huxley's new statements, the matter appeared in quite a new light—the more as I could never succeed in discovering a trace of a nucleus in an enamel prism. Huxley, in fact, asserts that the enamel is formed beneath the membrana preformativa, and that the membrana preformativa and cuticle of the enamel are identical. In this point—as I have found by examining the fresh teeth of a new-born child and those of a six months' fœtus—he is about right."

I wish I could give the entire force of M. Lent's exquisite and polite acknowledgment of the truth of a fundamental fact for all further theories of dental development, but here is the original, for those who can appreciate it: "*Hiermit hat es seine Richtigkeit.*"

In this part of M. Lent's paper a second misstatement occurs, somewhat more gross, if possible, than that which I have already had occasion to notice.

At page 131 he says: "Of nuclei, such as Huxley describes and figures (in the membrana preformativa,) I have seen nothing."

I have carefully re-examined my own paper, to see if I could find any excuse for a statement so utterly contrary to fact as this; and, for the sake of MM. Lent and Kölliker, I really almost reget to say I can find none whatever.

I invariably call the membrana preformativa a *structureless membrane.* I state that Nasmyth's membrane is "about $\frac{1}{2500}$ to $\frac{1}{1600}$ inch thick, perfectly clear and transparent, and, under a high power, exhibits innumerable little ridges upon its outer surface, which bound spaces sometimes oval and sometimes quadrangular, and about $\frac{1}{5000}$ of an inch in diameter." And I go on to say that this membrane is nothing but the altered structureless membrana preformativa. I am equally at a loss to discover any figures of these "nuclei;" though it is possible that in consequence of the reticulation not having been carried evenly all over Nasmyth's membrane in fig. 2, a very careless person might misunderstand the figure—the text, however, would at once correct this misapprehension.

I have neither space nor inclination to follow M. Lent further; as what has been said proves abundantly the only point worth discussing at all: viz. that the two main facts asserted in my paper are admitted to be *bonâ fide* additions to our knowledge; in fact, one might almost fancy M. Kölliker addressing to M. Lent, Balak's famous reproach to Balaam—"I called upon thee to curse this people, and, lo! thou hast blessed them these many times."

So far as I am personally concerned, I care little enough about being absorbed, after Professor Kölliker's ordinary fashion, in the next addition of the "Mikroskopische Anatomie," where we shall, I doubt not, read, "I and Huxley have made out that the enamel is formed under the membrana preformativa," &c. &c. But I think that Professor Kölliker will do well to reflect whether he is likely to increase his most deservedly high reputation, by encouraging in a student a disengenuousness of which he himself, I hope, would be heartily ashamed.

I may add, in conclusion, that there are, I believe, two very good minor grounds of cavil in my paper. One is at page 159, where I state incidentally that Professor Kölliker does not mention Nasmyth's discovery in his "Mikroskopische Anatomie"—an error for which I cannot account, and for which I can only apologise, as a complete oversight. The other is the description of the cement of the calf's tooth at page 162; in which, a subsequent examination has led me to think there are errors of interpretation. I have had no leisure to re-examine this point, and I recommend it to MM. Lent and Kölliker, if, as it would seem, they have some unaccountable source of satisfaction in finding me wrong—a thing I do myself, quite without satisfaction, every day.—*Microscopical Journal.*

### EDITORIAL DEPARTMENT.

